# An unusual case of thrombotic storm in an amateur cricketer—a case report

**DOI:** 10.1186/s12245-023-00539-4

**Published:** 2023-10-02

**Authors:** Takshak Shankar, Nagasubramanyam Vempalli, Archana Bairwa

**Affiliations:** Department of Emergency Medicine, All India Institute of Medical Sciences, Rishikesh, India

**Keywords:** Thrombotic storm, Hyperhomocysteinemia, Anticoagulation

## Abstract

**Background:**

Thrombotic storm is a series of acute to subacute thrombotic events that evolve over a few days to weeks and result in progressive thromboses at multiple sites. There is often a predisposing event to thromboses, such as trauma or infections. Prompt initiation of anti-coagulation can prove life-saving in such patients.

**Case report:**

We describe a previously healthy young male who developed thromboses of the right axillary, brachial, radial, and ulnar arteries while bowling in a cricket match. A few hours later, he developed a stroke involving the right anterior and middle cerebral arteries. His thrombophilia workup was significant for elevated homocysteine levels. Although he had a delayed presentation to our hospital, he was treated with anticoagulation and given a trial of thromboembolectomy, which failed and he had to ultimately undergo a right below-elbow guillotine amputation.

**Conclusion:**

Thrombotic storm should be recognized promptly in the Emergency Department and timely anticoagulation should be initiated.

## Background

A thrombotic storm is an extreme prothrombotic state which can involve both arterial and venous circulation and may occur at unusual locations. These events happen over a short period of time, such as days or weeks. Without adequate anticoagulation therapy, the process may continue on its own and may even lead to death [1]. We present a case of a 26-year-old previously healthy male who developed a thrombotic storm while bowling in a cricket match.

### Case report

A 26-year-old male presented to the emergency department (ED) with altered mental status for 1 day. The patient developed an episode of profuse sweating and numbness of the right hand while bowling in a cricket match the day prior. He rested for some time and as the symptoms subsided, he continued to bowl. After he had bowled the next ball, he developed sudden onset severe pain of the right upper limb. He was rushed to a local doctor who referred him to the ED, after giving an analgesic. On the way to the hospital, around 4 h after the initial episode of pain, he developed slurring of speech which progressed to altered sensorium over the next hour. There was no history of fever, chest pain, abdominal pain, headache, or similar complaints in the past. The patient had no significant past medical or surgical history. There was no history of any drug abuse, smoking, or alcohol use. There was no significant family history.

On arrival, the patient has altered sensorium with a Glasgow Coma Scale of 11. (E 4; V 2; M 5). He was afebrile, pulse rate of 90 beats per min, regular, blood pressure of 150/80 mm of mercury, peripheral oxygen saturation of 95% on room air, respiratory rate of 24 breaths per minute. His right upper limb was cool to touch and had absent brachial, radial, and ulnar pulses. Other peripheral pulses were palpable. On central nervous system examination, his pupils were equal bilaterally, 2 mm, with normal reaction to light. The tone in the left upper limb and lower limb was reduced, and there was a paucity of movement of the right upper limb. The plantar reflex was extensor on the left side and flexor on the right side. Meningeal signs were absent. The rest of the systemic examination was within normal limits.

**Fig. 1:**
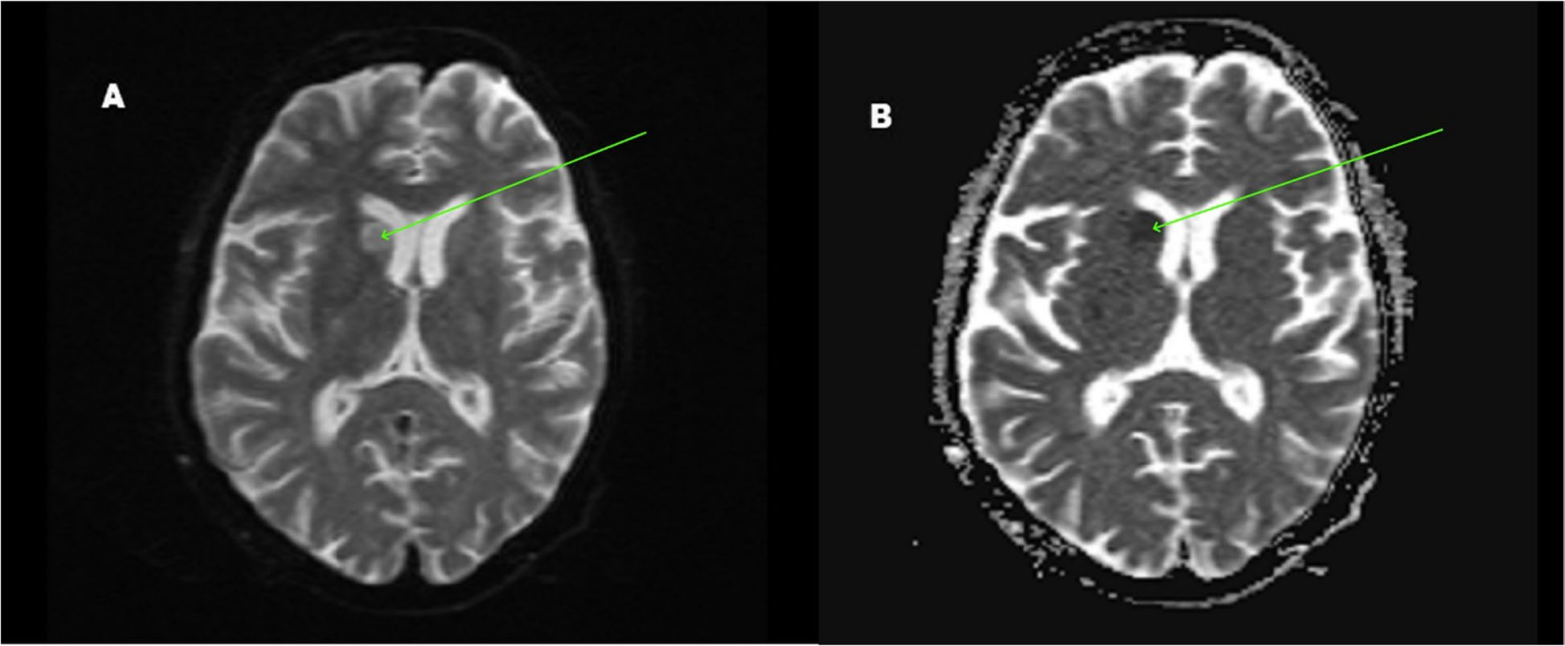
**A** Diffusion-weighted magnetic resonance imaging axial section of the brain showing restricted diffusion in the head of the caudate nucleus (green arrow). **B** Apparent diffusion coefficient (ADC) magnetic resonance imaging axial section of the brain showing low ADC values in the head of the caudate nucleus (green arrow)

**Fig. 2:**
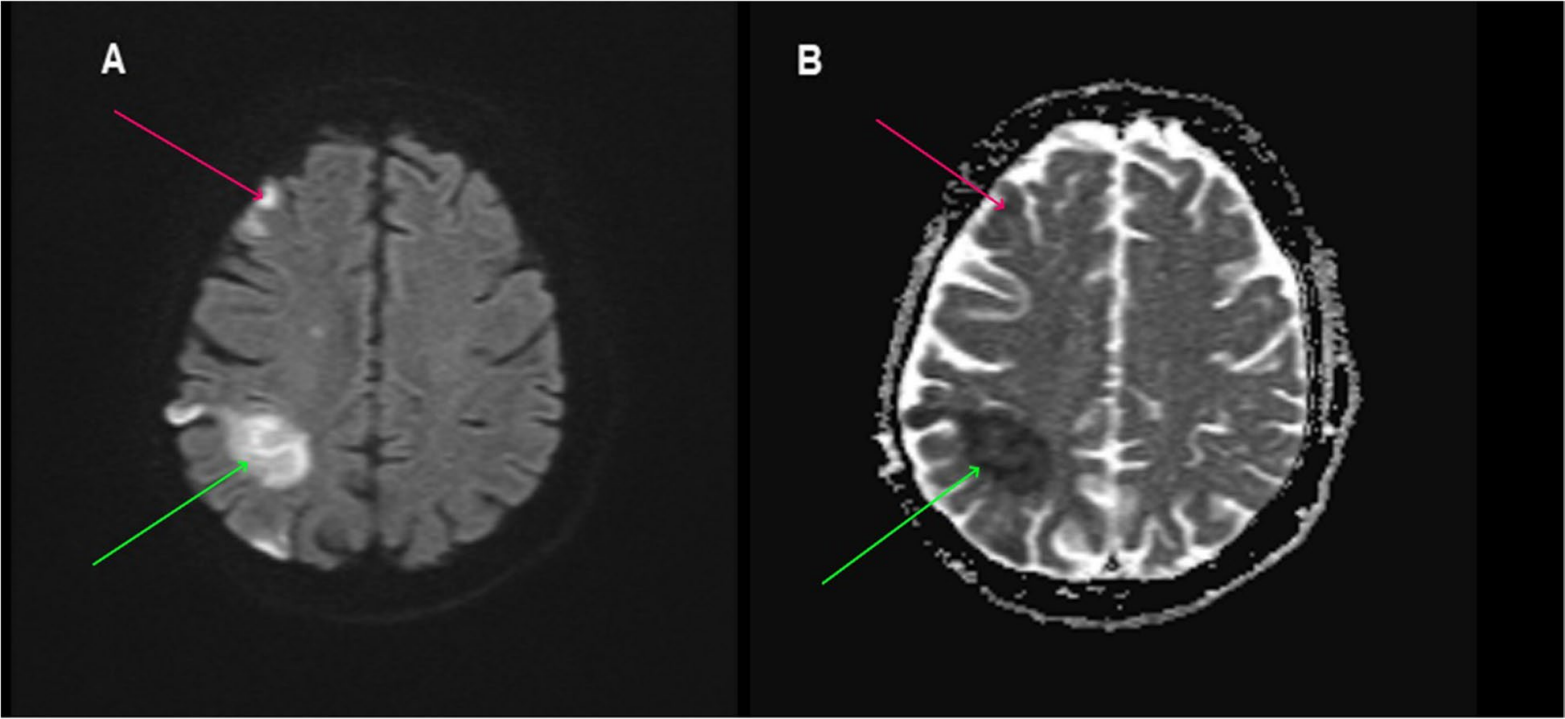
**A** Diffusion-weighted magnetic resonance imaging axial section of the brain showing restricted diffusion in the right frontal lobe (red arrow) and right parietal lobe (green arrow). **B** Apparent diffusion coefficient (ADC) magnetic resonance imaging axial section of the brain showing low ADC values in the right frontal lobe (red arrow) and right parietal lobe (green arrow)

Initial laboratory investigations sent from the emergency revealed that the patient’s hemoglobin, platelet count, creatinine, international normalized ratio, activated partial thromboplastin time, and serial Troponin I were within normal limits. The patient’s urine one-step immunochromatography was negative for D-amphetamine, Barbiturates, Benzodiazepines, Cocaine, Opiates, and Marijuana. *Electrocardiogram* was suggestive of a normal sinus rhythm. Two-dimensional echocardiography was within normal limits with a left ventricular ejection fraction of 60%, normal valves, and no clot or vegetation. A color Doppler ultrasound of the right upper limb demonstrated echogenic thrombus in the axillary, brachial, radial, and ulnar artery with absent blood flow along with inaudible venous Doppler signals. An ultrasound arterial and venous doppler of the left upper limb and lower limb was normal. A magnetic resonance imaging brain-stroke protocol revealed areas of restricted diffusion with corresponding low apparent diffusion coefficient values involving head of right caudate nucleus, right corona radiata, right frontal, and parietal lobes suggesting an acute infarct (Figs. 1 and 2).

Thrombophilia work-up of the patient sent later after admission, was significant for intermediately elevated homocysteine levels (40.33 μmol/L, reference value 5–15 μmol/L). The work-up for anticardiolipin IgG and IgM antibodies, prothrombin G20210A mutation, factor V Leiden mutation, and lupus anticoagulant was negative. Protein C activity, free protein S antigen, antithrombin III levels, ADAMTS13 levels, factor II levels, and factor VIII levels were normal.

The patient presented around 20 h after the onset of pain, with inaudible arterial and venous doppler, but some movement of the right upper limb. Due to his young age, with the dominant arm being involved, he was given a trial of right axillary and brachial thromboembolectomy for limb salvage by the vascular surgery team. In consultation with the vascular surgery team, it was decided to forgo a computed tomography angiography of the right upper limb and shift the patient directly to the operating room. The patient was shifted to the operating room within 1 h of Emergency arrival. Before the surgery, in consultation with the neurology team, the vascular surgery team, and with the consent of the caregivers, a bolus of unfractionated heparin 60 Units/kg was administered in the emergency. A good forward flow was achieved during the surgical procedure, but the backflow from the radial and ulnar arteries was not adequate. Following surgery, the patient was put on heparin infusion at 12 Units/kg/h with activated partial thromboplastin time monitoring. The patient was also started on aspirin 150 mg once daily, nicorandil 5 mg twice daily, vitamin B complex supplementation, and other supportive treatment in the form of wound care and passive limb movements. However, the patient’s gangrene progressed, and he had to undergo right below elbow guillotine amputation. Ultimately, the patient expired from refractory septic shock from ventilator-associated pneumonia.

## Discussion

Cricket is a very popular sport in India. Bowling in cricket involves overhead throwing of the bowl. This overhead throwing action involves 90 to 120° of shoulder abduction, full wrist pronation, and excessive external arm rotation [2]. This can lead to compression of the third part of the axillary artery by the anterior displacement of the humeral head. This occurs because the third part of the axillary artery is relatively fixed at its site by the overlying fascia, pectoralis minor muscle, and branch vessel origins. Repetitive positional compression of the axillary artery can result in lesions such as focal intimal hyperplasia, aneurysm formation, and segmental dissection. These lesions can contribute to thrombosis and distal embolism. These lesions have also been reported in baseball pitchers and volleyball players [3, 4].

A thrombotic storm is a prothrombotic event, defined based on its characteristic clinical behavior. Multiple thrombotic events, which can involve both arterial and venous circulation, occur in diverse vascular beds in a short period. Thrombotic storm usually occurs in individuals younger than 55 years of age and is frequently preceded by a trigger. The triggering factors can be pregnancy, surgery, trauma, infections, and inflammatory states. Anticoagulation is the primary treatment for patients diagnosed with a thrombotic storm. It should be initiated early and the therapeutic level of anticoagulation should be attained as quickly as possible, as subtherapeutic levels are associated with an increase in thromboembolic complications. If the thrombotic cycle is interrupted early, long-term prognosis is good [1, 5, 6].

Hyperhomocysteinemia is also associated with arterial occlusive disease [7–10]. It can result from both genetic and acquired conditions. Specific therapy for hyperhomocysteinemia includes pyridoxine, folic acid, and hydroxycobalamine [11].

Emergency management of patients with thrombotic storm should focus on prompt initiation of adequate anticoagulation. Additionally, a rapid search for the underlying etiology should also be carried out, so that targeted treatment could be started. This phenomenon has been described in various diseases such as antiphospholipid syndrome, thrombotic thrombocytopenic purpura and of late, coronavirus disease-2019 patients. In some patients, however, no underlying etiology is found. Although there is a lack of clinical trials, thrombolytic therapy could be considered for patients presenting with massive pulmonary embolism, extensive deep vein thrombosis, acute stroke, or peripheral arterial occlusion, with a preference for catheter-directed thrombolytic therapy where feasible. Antiplatelet therapy could also be used along with anticoagulant therapy in patients with arterial thromboembolic events. Adequate management of these patients requires a multidisciplinary support [12, 13].

Our patient was a young, previously healthy male who developed thrombosis of the right upper limb arteries while bowling in a cricket match, which progressed to a stroke. He was an amateur cricketer. After ruling out cardiac causes in our patient, the condition was attributed to thrombotic storm. Repeated positional compression of the axillary artery while bowling, in a background of hyperhomocysteinemia, likely triggered the thrombotic storm in our patient. He was started on anticoagulation and was given a trial of revascularization surgery for limb salvage. Post-surgery, he was put on anti-platelets and hyperhomocysteinemia therapy in addition to anticoagulant therapy. He was also prescribed tablet Nicorandil, as it causes both arterial and venous vasodilation through both nitric oxide donation and by opening adenosine triphosphate-dependent potassium channels [14]. It is crucial to recognize this condition in a timely manner in the emergency department, so that anticoagulation can be promptly initiated.

## Conclusion

Thrombotic storm is a life-threatening emergency whose diagnosis relies mostly on the clinical presentation of the patient. Early initiation of appropriate and adequate anticoagulation can prove life-saving in such patients. Subtherapeutic anticoagulation may lead to the progression of thrombosis. It is thus, crucial, that this condition be recognized promptly in the emergency department, so that timely treatment can be initiated.

## Data Availability

Not applicable.

## Abbreviations

ED: Emergency department
ADC: Apparent diffusion coefficient
